# Inhibition of proliferation and induction of differentiation of glioma cells with *Datura stramonium* agglutinin

**DOI:** 10.1038/sj.bjc.6600550

**Published:** 2002-10-07

**Authors:** T Sasaki, K Yamazaki, T Yamori, T Endo

**Affiliations:** Department of Glycobiology, Tokyo Metropolitan Institute of Gerontology, 35-2 Sakaecho, Itabashi-ku, Tokyo 173-0015, Japan; Division of Molecular Pharmacology, Cancer Chemotherapy Center, Japanese Foundation for Cancer Research,1-37-1 Kami-Ikebukuro, Toshima-ku, Tokyo 170-8455, Japan

**Keywords:** glioma, DSA, glycan, differentiation

## Abstract

We found that a lectin, *Datura stramonium* agglutinin, induced irreversible differentiation in C6 glioma cells. The differentiated cells had long processes, a low rate of proliferation and a high content of glial fibrillary acidic protein. When the medium was replaced with *Datura stramonium* agglutinin-free medium after 1 h, cell proliferation continued to be inhibited. Experiments with several other lectins indicated that both recognition of linear *N-*acetyllactosamine repeats and recognition of multiantennary units of cell-surface glycans were required for the inhibition of C6 proliferation. Proliferation of four human glial tumour cells was also inhibited by *Datura stramonium* agglutinin. Further, these differentiated human glial tumour cells had long processes and a high content of glial fibrillary acidic protein similar to differentiated C6 glioma cells. Taken together, these observations suggest that *Datura stramonium* agglutinin may be useful as a new therapy for treating glioma without side effects.

*British Journal of Cancer* (2002) **87**, 918–923. doi:10.1038/sj.bjc.6600550 www.bjcancer.com

© 2002 Cancer Research UK

## 

Malignant gliomas remain one of the most incurable forms of cancer in humans. While surgical and radiotherapeutic techniques have improved recently, the prognosis for patients with glial tumours still remains poor. Glial tumours are generally very highly malignant and infiltrate widely along axons. Most gliomas show indistinct boundaries between tumour tissue and normal tissue. Diffuse infiltration of the adjacent brain structures is the main reason that surgical resection is often incomplete, and even hemispherectomy may not prevent glioma recurrence (Burger *et al*, 1991). In the cases of tumours in other organs, malignant cells have been removed by surgical techniques, brachytherapy, and chemotherapy. In the case of brain tumours, however, extermination of malignant cells with any one of these treatments will cause extensive damage to normal cells and brain function, which explains why they are rarely curable (Black, 1991).

Since immature normal cells and malignant cells share similar characteristics, e.g. rapid proliferation, cellular migration and invasion, Linskey and Gilbert (1995) proposed that oncogenesis was a process of dedifferentiation or uncontrolled differentiation. According to this view, knowledge and control of normal cellular differentiation could help to understand and control malignancy. Differentiation agents may provide a means of leading tumour cells to re-differentiate, and thereby suppress tumour cells with minimal toxicity to normal cells. However, differentiation agents that have been investigated so far, such as retinoic acid, have not yet proved useful in treating gliomas.

One of the most important findings in the past decade in developmental biology is that there are well-controlled variations of oligosaccharides during development and differentiation. The glycan structures of tumour cells are almost always different from those of normal cells (Hakomori, 1985). Glycosylation of proteins is one of many molecular changes that accompany malignant transformation. During a malignant transformation, there are increases in the amounts of highly branched *N-*glycans and poly-*N-*acetyllactosamine chains (Dennis *et al*, 1999). In addition, terminal Lewis antigen sequences on highly branched *N-*glycans have been observed to increase in some cancers, and to correlate with poor prognosis (Dennis *et al*, 1999). Therefore, such tumour-specific glycans might be new molecular targets for treating glioma. Since some plant lectins are known to mimic natural biological response modulators, we examined a panel of plant lectins and found one that bound to tumour-specific glycans. It is *Datura stramonium* agglutinin (DSA) which binds specifically to glycans containing multiantennary and/or *N-*acetyllactosamine repeat units (Cummings and Kornfeld, 1984; Yamashita *et al*, 1987). Recently, we found that DSA induced differentiation of astrocytes (Sasaki and Endo, 2000). Addition of DSA caused a morphological change from a polygonal shape to a stellate shape with many long processes, an increase of glial fibrillary acidic protein (GFAP) and suppression of proliferation. Therefore, it was of interest to determine whether DSA can inhibit cell-specific proliferation and induce differentiation of glioma cells.

In the present study, we examined whether DSA induced differentiation of C6 cells and human glioma cell lines into the mature astrocytic phenotype and whether use of this lectin could provide a new approach to treating glioma.

## MATERIALS AND METHODS

### Reagents

*Datura stramonium* agglutinin (DSA) and tomato lectin (TL) were obtained from Sigma (St. Louis, MO, USA). *Dolichos biflorus* agglutinin (DBA), *Sambucus sieboldiana* agglutinin (SSA), leucoagglutinin from *Phaseolus vulgaris* (L-PHA), and *Lotus tetragonolobus* agglutinin (LTA) were obtained from Seikagaku Corp. (Tokyo, Japan). Kanamycin sulphate, Dulbecco's modified Eagle's medium (DMEM), RPMI 1640 and foetal bovine serum (FBS) were obtained from GIBCO BRL (Grand Island, NY, USA). Phenylmethylsulphonyl fluoride (PMSF), leupeptin hemisulphate, aprotinin, bovine serum albumin (BSA), 3,3′-diaminobenzidine (DAB) and pepstatin A were obtained from Nacalai Tesque (Kyoto, Japan). The BCA protein assay kit was purchased from Pierce Chemical Company (Rockford, IL, USA). Polyvinylidene difluoride (PVDF) membrane was obtained from Millipore Corporation (Bedford, MA, USA). Peroxidase-conjugated avidin (Vectastain ABC kit) was purchased from Vector Laboratories Inc. (Burlingame, CA, USA). Anti-glial fibrillary acidic protein (GFAP) antibody was obtained from DAKO Corp., (Carpinteria, CA, USA). The peroxidase-linked F(ab′)_2_ fragment of anti-rabbit IgG was obtained from Amersham Pharmacia Biotech Inc., (Piscataway, NJ, USA).

### Cell cultures

The rat glioma cell line C6, was obtained from Dr K Watanabe (our institute). Cells were cultured in DMEM supplemented with 10% FBS, 0.1 mg ml^-1^ kanamycin, and 4 mg ml^−1^ glucose at 37°C under a humidified atmosphere of 5% CO_2_ −95% air. Human brain tumour-cell lines, U251 (glioblastoma), SF-539 (gliosarcoma), SNB-75 (astrocytoma), and SNB-78 (astrocytoma) were cultured as described previously (Monks *et al*, 1991; Yamori *et al*, 1999).

### Morphological observations

Changes in cell morphology were assessed under a microscope (TE300, Nikon Co. Ltd., Tokyo, Japan) with a Hoffman Modulation Contrast module (Nikon Co. Ltd.). C6 cells and human glial tumour-cell lines were seeded in 12-well plates (Asahi Techno glass) at a density of 1.0×10^4^ cells per cm^2^. Cells were incubated with 1 μM DSA or without DSA beginning 1 h after seeding. After 23 h, photographs of each cell were taken. Experiments were performed more than five times.

### Cell proliferation assay

Plated cell numbers per well in 12-well plates of C6 cells and human tumour-cell lines (U251, SNB-75, and SNB-78) were 0.5×10^4^, 0.4×10^4^, 1.0×10^4^, and 1.0×10^4^ cells, respectively. Five wells were used for each experimental condition. Cells were detached with trypsin and EDTA and counted in a Bürker-Türk haemocytometer. Values were expressed as the mean±standard deviation.

DNA synthesis was measured as the amount of 5′-bromodeoxyuridine (BrdU) incorporated using ‘Cell Proliferation ELISA, BrdU (colorimetric)’. The subcultured astrocytes were seeded at a density of 1.9×10^4^ cells per cm^2^ in a 96-well plate (Nippon Becton Dickinson Co., Ltd., Tokyo, Japan).

Cell growth inhibition (percentage of growth) was calculated as:

% growth=100×{(*T* – *T*_0_)/(*C* – *T*_0_)}

where *C* and *T* are the cell number of the control and test wells, respectively, and *T*_0_ is the cell number immediately before the addition of DSA.

### SDS–PAGE and immunoblotting

The cells in the culture dish were washed three times with ice-cold PBS, detached in ice-cold PBS with a rubber scraper, collected by centrifugation at 600 *g* for 10 min at 4°C, and homogenised with 1% SDS in 10 mM Tris-HCl buffer, pH 7.4, containing 1 mM PMSF, 10 μM leupeptin, 1 μM aprotinin, 1 μM pepstatin A and 1 mM EDTA.

SDS–PAGE (10% acrylamide) was performed essentially as described by Laemmli (Laemmli, 1970). Protein concentration was determined with a BCA protein assay kit. Each sample (10 μg from C6 or 5 μg from human tumour-cell line) was subjected to SDS–PAGE. For the immunoblot, the proteins in the gel were transferred electrophoretically to a PVDF membrane at 2 mA/cm^2^ for 2 h in 25 mM Tris, 192 mM glycine and 20% methanol, pH 8.3, using a protein transfer system (Bio-Rad Laboratories, Hercules, CA, USA). The PVDF membrane was incubated with PBS containing 3% BSA at 4°C for about 12 h, incubated with a solution containing the anti-GFAP polyclonal antibody in PBS containing 1% BSA at room temperature for 2 h with shaking, incubated with an anti-rabbit IgG antibody conjugated with peroxidase in PBS containing 1% BSA for 1 h, washed three times with PBS containing 0.05% Tween 20 for 5 min, washed once with PBS for 10 min, and incubated with the peroxidase substrate, DAB, to detect reactive proteins. Band intensities were measured by densitometric scanning using a densitometer and NIH Image 1.61/ppc software.

## RESULTS

### Effect of DSA on the proliferation of C6 cells and human glial tumour-cell lines

**Figure 1:**
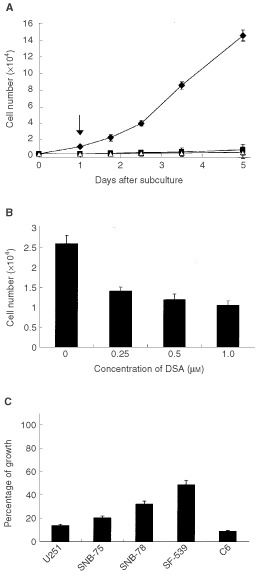
Inhibition of proliferation of glioma cells by DSA. (**A**), Effect of DSA on proliferation of C6 cells. Triangle, Cells exposed to 1 μM DSA after the seeding; diamond, control cells; square, cells after DSA-containing medium was replaced with DSA-free medium. Arrow indicates time of change of medium. Cells were detached with trypsin and EDTA and counted in a Bürker-Türk haemocytometer at the indicated times after the seeding. C6 cells were seeded in 12-well plates (Asahi Techno glass) at a density of 0.5×10^4^ cells per well and cultured in 10% FBS-supplemented media. The data are presented as mean±s.d.; *n*=5 for each experimental condition. (**B**), Concentration-dependence of inhibition of proliferation of C6 cells. C6 cells were seeded in 12-well plates at a density of 1.0×10^4^ cells per well and cultured in 10% FBS-supplemented media. DSA was added 3 h after the seeding of the cells at the indicated concentrations. Cell numbers were counted 24 h after seeding. The data are presented as mean±s.d.; *n*=5 for each DSA concentration. (**C**), Effect of DSA on proliferation of four human glial tumour cells. DSA was added 24 h after seeding of the cells at a concentration of 1 μM. After 1 h exposure with DSA in FBS-free medium, the medium was changed to DSA-free, FBS-supplemented medium. After 36 h culture, cell growth was measured. Data are expressed as a percentage of growth as described in Materials and Methods.

To examine the effects of DSA on glioma cell proliferation, cell numbers were counted after the addition of DSA. One μM of DSA almost completely inhibited the proliferation of C6 cells (Figure 1A

). After 120 h, the number of control C6 cells increased 28.9 times (Figure 1A, diamonds), but DSA-treated cells increased only 1.14 times (Figure 1A, triangles). DSA suppressed C6 proliferation in a dose-dependent manner (Figure 1B). After 24 h, the number of C6 cells cultured without DSA increased 2.6 times. The corresponding values of C6 cells treated with 250 nM, 500 nM, and 1 μM of DSA were 1.4, 1.2, and 1.1, respectively. We confirmed the results by a different method. Since 5′-bromodeoxyuridine (BrdU) incorporation of astrocytes cultured with 1 μM of DSA containing medium during 12 h culture was reduced to 4.3% in comparison with DSA free control, we concluded that DNA synthesis was strikingly suppressed by DSA.

After 24 h exposure to DSA, the medium was replaced with DSA-free medium. Despite the removal of DSA, the cells continued to proliferate at the same slow rate (Figure 1A, squares). Thus, the effect of DSA on C6 proliferation was irreversible and not a temporal response. These results suggested that DSA acted at an early stage of cell proliferation.

To determine whether the inhibitory effect of DSA on cell proliferation is limited to C6 cells, we examined the effect of DSA on the proliferation of four human glial tumour cells (U251, SF-539, SNB-75, and SNB-78). Exposure of each of these cells type to 1 μM of DSA for 1 h reduced their growth rate to 14% ∼ 49% of that of the control (Figure 1C). Under these conditions, growth rate of C6 cells reduced by over 90%. These results suggested that DSA was effective on all types of glioma cells.

### Induction of morphological changes of gliomas by DSA

**Figure 2:**
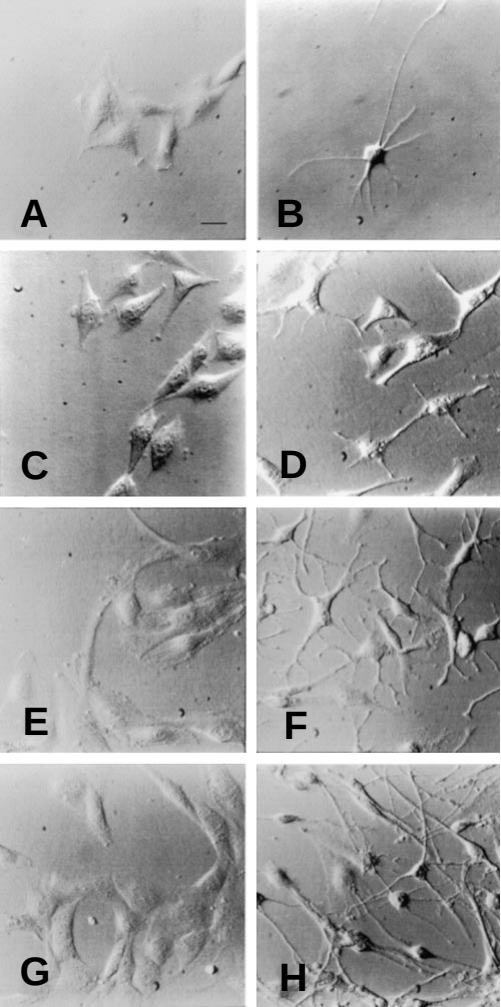
Morphological changes of glioma cells by DSA. Plated cell numbers per well of C6 cells and human tumour-cell lines (U251, SNB-75, and SNB-78) were 1.0×10^4^, 0.7×10^4^, 1.4×10^4^, and 1.4×10^4^ cells, respectively. C6 cells were cultured in 10% FBS-supplemented DMEM and human tumour-cell lines were done in 5% FBS-supplemented RPMI 1640. Cells were incubated without (**A**, **C**, **E**, and **G**) or with (**B**, **D**, **F**, and **H**) 1 μM DSA beginning 1 h after seeding. Cells were observed 23 h after the addition of DSA. (**A** and **B**), C6; (**C** and **D**), U251; (**E** and **F**), SNB-75; (**G** and **H**), SNB-78. Bar in (**A**), 50 μm.

To assess the morphological effects of DSA, we analysed C6 cells treated with or without DSA by microscopy (Figure 2A,B

). C6 cells had a flat epithelioidal morphology (Figure 2A), but after addition of DSA, most of them became astrocytic with many long, thin processes well-developed and extended radially (Figure 2B). The lengths of some processes exceeded 200 μm. In the absence of DSA, most cells were flattened and spindle-shaped and none of them had thin process (Figure 2A). Since it is known that well-differentiated astrocytes have a stellate shape and many long processes (Butt, 1991), the present results suggests that DSA caused C6 cells to enter the differentiated state.

When we examined the effect of DSA on the morphological changes of human glial tumour cells, most of them changed their shape as follows. U251 cells were a flat epithelioidal morphology (Figure 2C). After addition of DSA, most of them changed to a stellate shape (Figure 2D). In the case of SNB-75, the cells were elongated in comparison with C6 or U251 but flattened (Figure 2E). After addition of DSA, they changed to a stellate shape with well-branched processes (Figure 2F). The morphology of SNB-78 cells was similar to that of SNB-75 before addition of DSA (Figure 2G), but SNB-78 cells changed differently to a stellate shape with longer but fewer branched processes than SNB-75 cells after addition of DSA (Figure 2H). These results indicated that DSA induced the morphological changes of glial tumour cells from the flatten epithelioidal shape to the stellate form bearing two or more thin processes. It has been reported that such a stellate cell morphology is one of characteristics of differentiated astrocytes (Bovolenta *et al*, 1984; Butt, 1991).

### Increased expression of GFAP after addition of DSA

**Figure 3:**
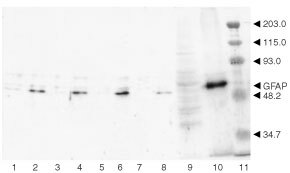
Immunoblotting with anti-GFAP antibody. Cells cultured in media containing 10% FBS, were collected 24 h after incubation without (lanes 1, 3, 5, 7 and 9) and with DSA (lanes 2, 4, 6, 8 and 10). Lanes 1 and 2, U251; lanes 3 and 4, SNB-75; lanes 5 and 6, SNB-78; lanes 7 and 8, SF-539; lanes 9 and 10, C6. SDS-solubilised fractions obtained from each cell preparation were applied to SDS–PAGE using a 10% acrylamide gel, and the separated proteins were transferred to a PVDF membrane. Lanes from 1 to 8 contained 5 μg protein, and lanes 9 and 10 did 10 μg. The PVDF membrane was stained with anti-GFAP antibody. The migration positions of GFAP and molecular weight markers (kilodalton) are indicated (lane 11).

The malignancy of gliomas is inversely correlated with the content of astrocyte-specific intermediate filament protein (GFAP) (Tascos *et al*, 1982). Furthermore, mature astrocytes are characterised by the expression of a large amount of GFAP (Tascos *et al*, 1982; Chiu and Goldman, 1985). GFAP is expressed exclusively in astrocytes and the expression level increases during differentiation. C6 cells were found to produce only a small amount of GFAP due to their malignant properties (Messens and Slegers, 1992). The immunoblot in Figure 3

shows clearly that the expression of GFAP was enhanced after the addition of DSA. A densitometric evaluation of the blot indicated that the amount of GFAP after the addition of DSA increased over hundred fold relative to the amount in the control cells. The corresponding values of human glial tumour cell lines were more than 12.2 times. Differentiated astrocytes are characterised by a stellate cell morphology, increased GFAP expression, and inhibited proliferation (Butt, 1991; Bovolenta *et al*, 1984). We conclude that C6 cells and human glial tumour-cell lines were induced to differentiate by DSA.

### Lectin specificity of inhibitory effect on C6 proliferation

**Figure 4:**
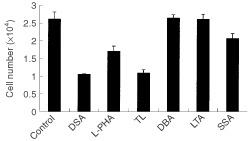
Lectin specificity of inhibitory effect on C6 proliferation. C6 cells were seeded in 12-well plates at a density of 1.0×10^4^ cells per well and cultured in 10% FBS-supplemented media. One μM of each lectin was added 3 h after the seeding of the cells. Cell numbers were counted 24 h after seeding. Data are presented as mean±s.d.; *n*=5 for each lectin. Twenty-four hours after seeding, the number of C6 cells cultured in DSA-free medium increased 2.64 times (control). The corresponding values of cells treated with DSA, L-PHA, TL, DBA, LTA and SSA were 1.06, 1.71, 1.09, 2.65, 2.61 and 2.06, respectively.

DSA binds specifically to glycans containing multiantennary and/or *N-*acetyllactosamine repeat units (Cummings and Kornfeld, 1984; Yamashita *et al*, 1987). Therefore, it was of interest to determine whether other plant lectins, which possess different carbohydrate-binding specificities, can inhibit proliferation of C6 cells. The results are shown in Figure 4

. TL, which reacts with a linear *N-*acetyllactosamine repeat sequence of three or more units (Kilpatrick *et al*, 1984; Merkle and Cummings, 1987), inhibited C6 proliferation as strongly as DSA. L-PHA which recognises multiantennary *N-*glycans containing a Galβ1→4GlcNAcβ1→6(Galβ1→4GlcNAcβ1→2)Man group (Cummings and Kornfeld, 1982), was also effective. These results indicate that the presence of both linear *N-*acetyllactosamine repeats and multiantennary units may be required for the inhibition of C6 proliferation. It is noteworthy that DBA, which binds to core 1 *O-*linked oligosaccharide (Galβ1→3GalNAc) (Baker *et al*, 1983), did not inhibit proliferation at all. LTA, a fucose-binding lectin (Allen *et al*, 1977; Petryniak *et al*, 1983), also did not inhibit proliferation. Since SSA showed only a small effect on C6 proliferation, glycans containing the Siaα2→6 group may not be effective (Shibuya *et al*, 1989). We conclude that DSA inhibited C6 proliferation in a glycan-specific manner, via glycans containing multiantennary and/or *N-*acetyllactosamine repeat units.

## DISCUSSION

During organogenesis in the central nervous system, differentiated astrocytes are characterised by a high expression of GFAP, a change from a polygonal shape to a stellate shape, and a low rate of proliferation (Wang *et al*, 1994). In the present study, we showed that DSA-treated cells express the characteristics of differentiation, i.e., they had a high content of GFAP, long processes, and a low rate of proliferation. GFAP is expressed exclusively in astrocytes, and that the amount of GFAP is closely related to the differentiation of astrocytes (Dahl, 1981; Bovolenta *et al*, 1984). In addition to being a marker of matured astrocytes, GFAP is considered to be important for the induction and maintenance of astrocyte differentiation, because introduction of GFAP to C6 cells and human astrocytoma induced morphological changes, and suppressed their proliferation and invasive potential (Rutka and Smith, 1993; Toda *et al*, 1994).

A strategy was proposed to control glial tumours by cellular differentiation (Linskey and Gilbert, 1995). Regulation of cell proliferation and terminal differentiation is a critical aspect of normal development and homeostasis (Raff, 1996), but is frequently disrupted during tumorigenesis (Sawyers *et al*, 1991). Thus, DSA may be useful for treating gliomas.

It is well known that neuronal cells, in addition to astrocytes, are important. Therefore, it is important to avoid any effects on neurons during treatment of glial tumours. It is desirable that the inhibitory effect of DSA on the proliferation of glial tumour cells continues after removal of DSA. Some agents that inhibit cell proliferation, such as dibutyryl cyclic AMP, have only a transient effect and thus must be continuously present. In contrast, DSA induced irreversible differentiation of tumour cells after a short exposure. Thus, DSA may be useful as an anti-tumour drug because such a short exposure may minimize the side effects on other normal cells. Treatment with DSA once or a few times may be enough to treat the tumour.

Many molecules on the surface of astrocytes including cell adhesion molecules and receptors are shared by neurons. Their effector molecules may bind to the molecules on both cells, and then they may affect not only astrocytes but also neurons. It is well known that the structures of glycans are specific for each cell type (Fukuda, 1985; Kagawa *et al*, 1988). Since lectins recognise specific sequences and configurations of the oligosaccharides, they are useful tools for distinguishing glycoproteins produced by different cells. In fact, DSA reacted strongly with many rat astrocyte glycoproteins, but reacted with very few neuronal glycoproteins (Sasaki and Endo, 1999). Actually, DSA did not affect neuronal cell migration or axonal extension and did not induce any morphological change of neurons (Sasaki and Endo, 2000). Taken together, these results suggest that DSA can distinguish between astrocytic and neuronal glycoreceptors. Previously, Jacobs and Lakes-Harlan (1997) reported that DSA is bound to glial cells and/or glia in the surrounding extracellular matrix but not to neuronal cells in the locust. These results support the hypothesis that DSA selectively binds to astrocytes, but not to neuronal cells. Therefore, the effect of DSA on astrocytes may be restricted to glia in the brain. It is of interest that DSA was effective not only on rat C6 cells but also on four human glial tumours.

We previously found that DSA induced astrocyte differentiation through tyrosine dephosphorylation, specifically a decrease in the extent of tyrosine phosphorylation of a 38-kDa protein (Sasaki and Endo, 2000). Further studies are needed to elucidate molecular mechanism by which DSA acts on glioma cells.

In summary, we showed that DSA induced differentiation and inhibited the proliferation of glioma cells. After addition of DSA to glioma cells, the cells grew at a much reduced rate, they changed from a flattened epithelioidal shape to a stellate shape having two or more long processes, so that some of the cells resembled normal fibrous astrocytes, and their content of GFAP was strikingly increased. These changes showed that DSA caused glioma cells to differentiate and thus may be useful as a new therapy for human glioma.
